# Conductivity reactivity index for monitoring of cerebrovascular autoregulation in early cerebral ischemic rabbits

**DOI:** 10.1186/s12938-023-01142-7

**Published:** 2023-08-09

**Authors:** Jia Xu, Haocheng Li, Gui Jin, Wei Zhuang, Zelin Bai, Jian Sun, Mingsheng Chen, Feng Wang, Xu Yang, Mingxin Qin

**Affiliations:** 1 College of Biomedical Engineering, Third Military Medical University (Army Medical University), Chongqing, China; 2 Department of Medical Engineering, General Hospital of Central Theater Command, Wuhan, China; 3 Department of Medical Service, General Hospital of Central Theater Command, Wuhan, China

**Keywords:** Cerebrovascular autoregulation, Magnetic induction phase shift, Cerebral blood flow, Conductivity reactivity index, Cerebral ischemia

## Abstract

**Background:**

Cerebrovascular autoregulation (CVAR) is the mechanism that maintains constant cerebral blood flow by adjusting the caliber of the cerebral vessels. It is important to have an effective, contactless way to monitor and assess CVAR in patients with ischemia.

**Methods:**

The adjustment of cerebral blood flow leads to changes in the conductivity of the whole brain. Here, whole-brain conductivity measured by the magnetic induction phase shift method is a valuable alternative to cerebral blood volume for non-contact assessment of CVAR. Therefore, we proposed the correlation coefficient between spontaneous slow oscillations in arterial blood pressure and the corresponding magnetic induction phase shift as a novel index called the conductivity reactivity index (CRx). In comparison with the intracranial pressure reactivity index (PRx), the feasibility of the conductivity reactivity index to assess CVAR in the early phase of cerebral ischemia has been preliminarily confirmed in animal experiments.

**Results:**

There was a significant difference in the CRx between the cerebral ischemia group and the control group (p = 0.002). At the same time, there was a significant negative correlation between the CRx and the PRx (r = − 0.642, p = 0.002) after 40 min after ischemia. The Bland–Altman consistency analysis showed that the two indices were linearly related, with a minimal difference and high consistency in the early ischemic period. The sensitivity and specificity of CRx for cerebral ischemia identification were 75% and 20%, respectively, and the area under the ROC curve of CRx was 0.835 (SE = 0.084).

**Conclusion:**

The animal experimental results preliminarily demonstrated that the CRx can be used to monitor CVAR and identify CVAR injury in early ischemic conditions. The CRx has the potential to be used for contactless, global, bedside, and real-time assessment of CVAR of patients with ischemic stroke.

## Background

Cerebrovascular autoregulation (CVAR) is a brain protective mechanism maintaining the constant cerebral blood flow (CBF) by quickly adjusting the cerebral vascular caliber when arterial blood pressure (ABP) or cerebral perfusion pressure (CPP) fluctuates [1–3]. CVAR dysfunction has been reported in patients with ischemic stroke [4] and may cause secondary complications and influence patient outcomes [5, 6]. Monitoring CVAR can help the clinic to manage blood pressure individually, prevent complications and furthermore predict the prognosis of ischemic stroke patients [7–9].

When normal homeostasis of CVAR is disturbed by pressure excitation, the responding cerebral blood flow usually adjusts to a new homeostatic state. The principle of CVAR assessment is based on the evaluation of this excitation-response process after disturbance to diagnose the ability of CVAR to maintain adequate and stable cerebral blood flow [10, 11]. This ability is described by some indices functioning with the relationship between the exciting blood pressure and the responding blood flow or blood volume [12]. Assessment dynamic CVAR requires monitoring the successive changes in CBF or cerebral blood volume (CBV).

For real-time bedside monitoring, various methods have been used in CVAR studies, and various corresponding assessment indices have been established, including intracranial pressure (ICP) monitoring, which is a routine technique in neurological intensive care units (NICUs) [13, 14]. Although there is currently no gold standard indicator for CVAR monitoring, ICP is commonly used as a comparison in research [15]. ICP monitoring has been used as a surrogate physical quantity for CBV. Pressure reactivity index (PRx), a moving correlation coefficient between spontaneous slow oscillations in ABP and corresponding response in ICP, has been established in clinical studies to assess CVAR [16, 17]. Negative PRx values indicate intact CVAR, and conversely, positive PRx values indicate impaired CVAR. The PRx proposed by Czosnyka and his colleagues has been supported by a variety of clinical and animal studies [18–22]. However, ICP monitoring requires surgical operations, which may cause complications [23, 24]. Moreover, PRx is rarely monitored in clinical for ischemic patients because invasive ICP probes are not routinely implanted in patients with cerebrovascular pathologies. Some non-invasive monitoring methods, such as transcranial Doppler (TCD) [25] and near-infrared spectroscopy (NIRS) [26], etc., have been investigated for CVAR assessment and have been tried with mixed results [27].

It is well known that electrical impedance and conductivity of tissues can be used to characterize their properties. Various dynamic CVAR challenges lead to physiological changes in the brain, characterized by changes in the composition and structure of the brain tissues, in particular changes in the relative volumes of blood, cerebrospinal fluid, intracellular fluid, and extracellular fluid. These changes affect the electrical properties of the whole brain. It has been proved that transcranial bioimpedance monitoring, potentially a substitute for ICP measurement, also captures slow oscillations of cerebral intra-arterial volume stimulated by ABP changes [28]. The correlation coefficient between ABP and transcranial bioimpedance could also be used to assess the reactivity of cerebral vasculature and determine the lower limit of autoregulation [29].

In bioimpedance measurements, contact electrodes are required to deliver micro-sensory currents and measure the associated voltages. Electromagnetic induction, which measures the electrical properties of bio-tissues using non-contact coils, is considered a valuable alternative to contact bioimpedance measurements, without current coupling between the electrode and the contact skin or tissue [30]. For electromagnetic induction measurement of the brain, the skull is not implied as shielding the magnetic field [31]. One of the electromagnetic induction methods is magnetic induction phase shift (MIPS) measurement, which could realize non-invasive, non-contact, depth, and bedside monitoring of whole brain electrical properties. The MIPS measurement has been applied to detect cerebral ischemia, cerebral edema, cerebral hemorrhage, and cerebral trauma on the principle that the obtained MIPS is proportional to the conductivity of the brain tissue in the field [32–35]. Previous studies have confirmed that the MIPS signal changes proportionally with CBV changes [36, 37], and the contactless electromagnetic induction measurement is sensitive enough to detect CVAR [38], but whether a novel index based on the MIPS method can be established to assess CVAR with spontaneous oscillations blood pressure requires further investigation.

In this study, we established a conductivity reactivity index (CRx) based on the correlation coefficient between spontaneous slow oscillations of ABP and the corresponding MIPS and compared CRx with intracranial pressure reactivity index in ischemia rabbits. The study aimed to verify the feasibility of the novel CRx to assess CVAR in the early phase of ischemia.

## Results

### Magnetic induction phase shift captures changes of CBV

**Fig. 1:**
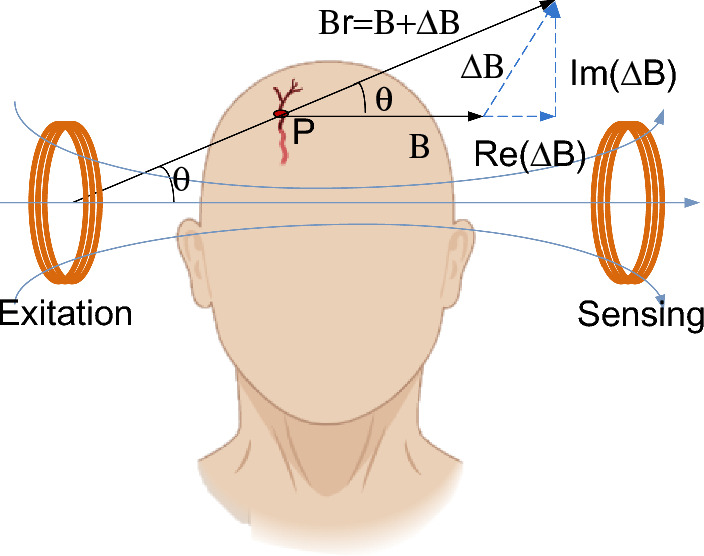
Magnetic field vector relation diagram of the MIPS detection

This study mainly applied MIPS as a substitute for CBV to detect CVAR. For MIPS detection, the measured biological tissue is placed between the excitation coil and the detection coil (Fig. 1). An alternating current in the excitation coil generates a sinusoidal alternating primary magnetic field (B). This primary magnetic field forms an induction current in the measured tissue and then creates an induced magnetic field (ΔB). Both the primary magnetic field and the induced magnetic field received by the detection coil are converted into an induced voltage. The vector (Br) at point P related to the induced magnetic field (ΔB) and the primary magnetic field (B) is shown in Fig. 1. According to Griffiths et al. [39, 40], if a sinusoidal signal with an angular frequency of ω is used for excitation, the magnetic vector potential follows:

1 $$\frac{\Delta B}{B} = Q\mu_{0} \omega \left( {\omega \varepsilon_{0} \varepsilon_{r} - {\text{j}}\sigma } \right)$$

Here, σ is the conductivity of the measured tissue, μ0 and ε0 are the vacuum permeability and the vacuum permittivity, respectively, εr is the relative permittivity of the measured tissue, and Q is a geometric constant relating to the location, structure and size of the measured tissues.

For biological tissues, since ΔB is much smaller than B, the MIPS, which is the phase angle $\Delta \theta$ between the induced magnetic field and the primary magnetic field, can be approximated as the imaginary part of ΔB/B [41]. When the frequency of excitation voltage is set, the MIPS is influenced by the conductivity σ of the bulk tissue:

2 $$MIPS = \Delta \theta \approx Q\omega \mu_{0} \Delta \sigma$$

Since brain tissues have different conductivities, the bulk conductivity σ of the brain alters with the relative volume changes of these tissues, which is reflected in the MIPS value.

### Preprocessing of the original signals (ABP, ICP, MIPS) for the CRx and the PRx

**Fig. 2:**
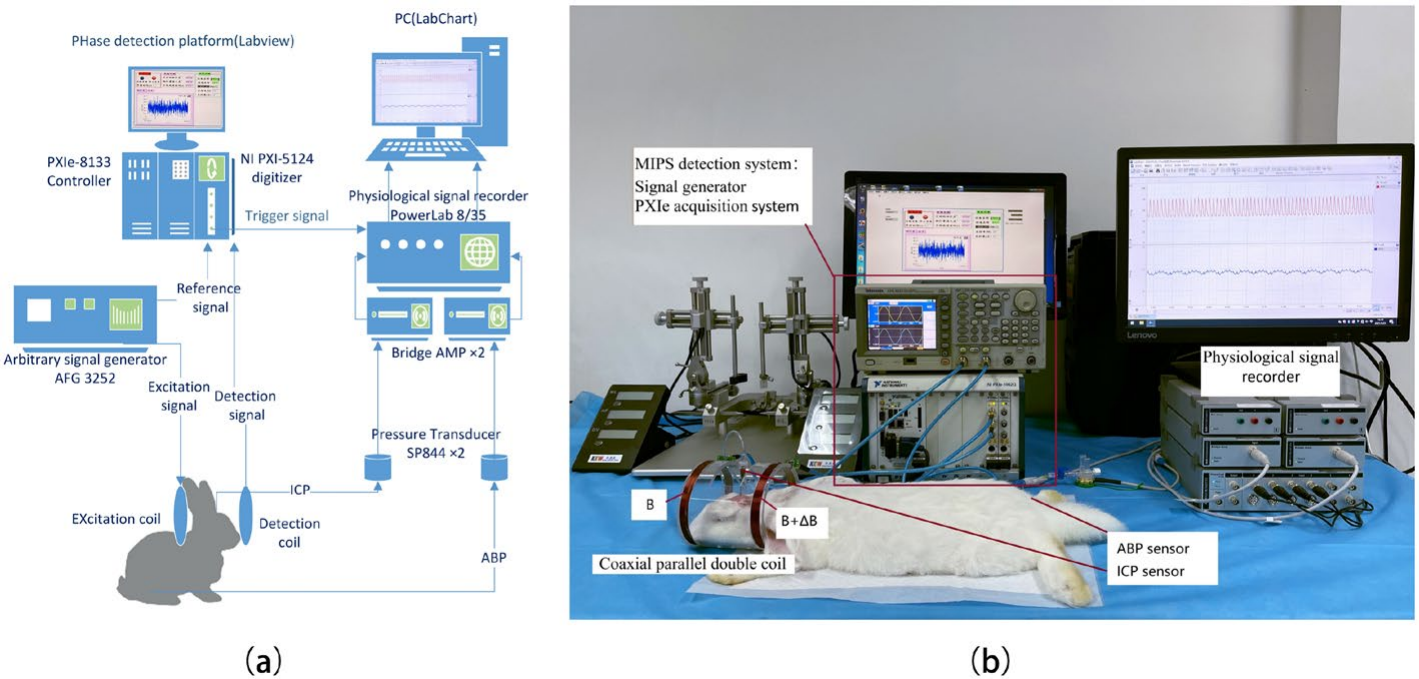
The experimental system of cerebrovascular autoregulation monitoring. **a** System block diagram. **b** Experiment on the cerebral ischemic rabbits and the MIPS sensor

To establish the CVAR index, ABP, ICP and MIPS of the were simultaneously monitored on the experiment rabbits, as shown in Fig. 2b. A moving Pearson correlation coefficient between ABP and MIPS was calculated to get the CRx, in the same way of PRx with ABP and ICP. The PRx and CRx were calculated as formula (3) and (4), respectively.

3 $$PRx = \frac{{{\text{cov}} (ABP,ICP)}}{{\sigma_{ABP}\,\sigma_{ICP} }}$$

4 $$CRx = \frac{{{\text{cov}} (ABP,MIPS)}}{{\sigma_{ABP}\,\sigma_{MIPS} }}$$

Here, cov is the covariance of the ABP and ICP/MIPS, $\sigma$ is the sample standard deviation of the ABP/ ICP/MIPS.

**Fig. 3:**
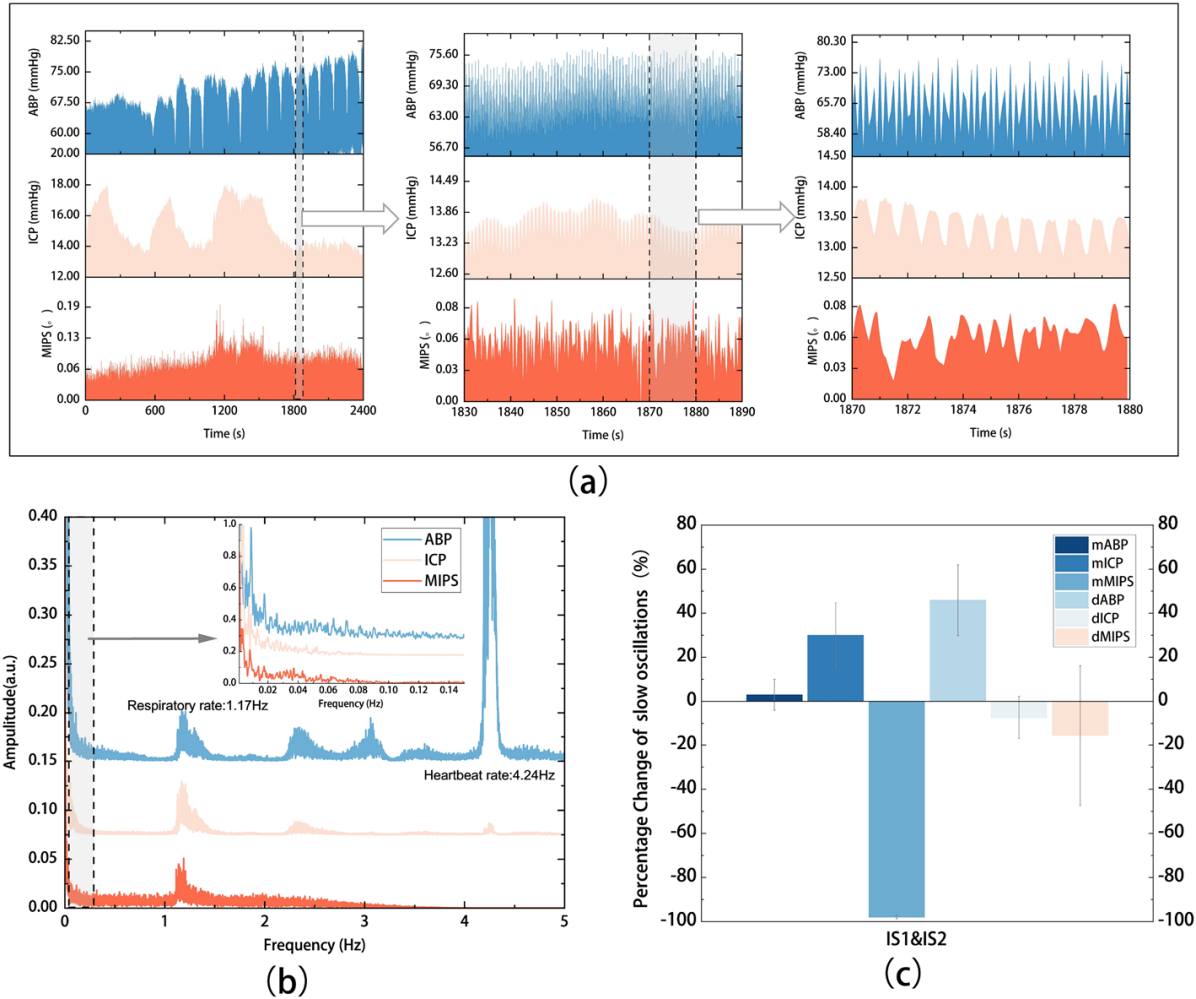
ABP, ICP and MIPS simultaneously monitored for CVAR assessment. **a** Time domain waveform and **b** frequency spectrum of ABP, ICP, MIPS in different time scales of ischemic rabbit (No. 7). **c** Mean values and amplitude oscillation ($\left|\mathrm{Vmax}-\mathrm{Vmin}\right|$) of slow oscillations wave in ABP, ICP and MIPS of the cerebral ischemia group

Figure 3a shows the original signals (ABP, ICP and MIPS) changed with the time at different scales. There were slow oscillations with inverse regularity in ICP and MIPS (Fig. 3a middle), which were different from the rate of respiration and heartbeat. The frequency spectrum of the three signals is shown in Fig. 3b. The respiratory rate is about 1.16 Hz, and heart rate is about 4.24 Hz. Respiratory components, were found in ABP, ICP and MIPS, and heartbeat components were found in ABP and ICP, which affect the CVAR index calculated with slow oscillations, especially in the MIPS which is completely submerged by respiratory (Fig. 3a right).

**Fig. 4:**
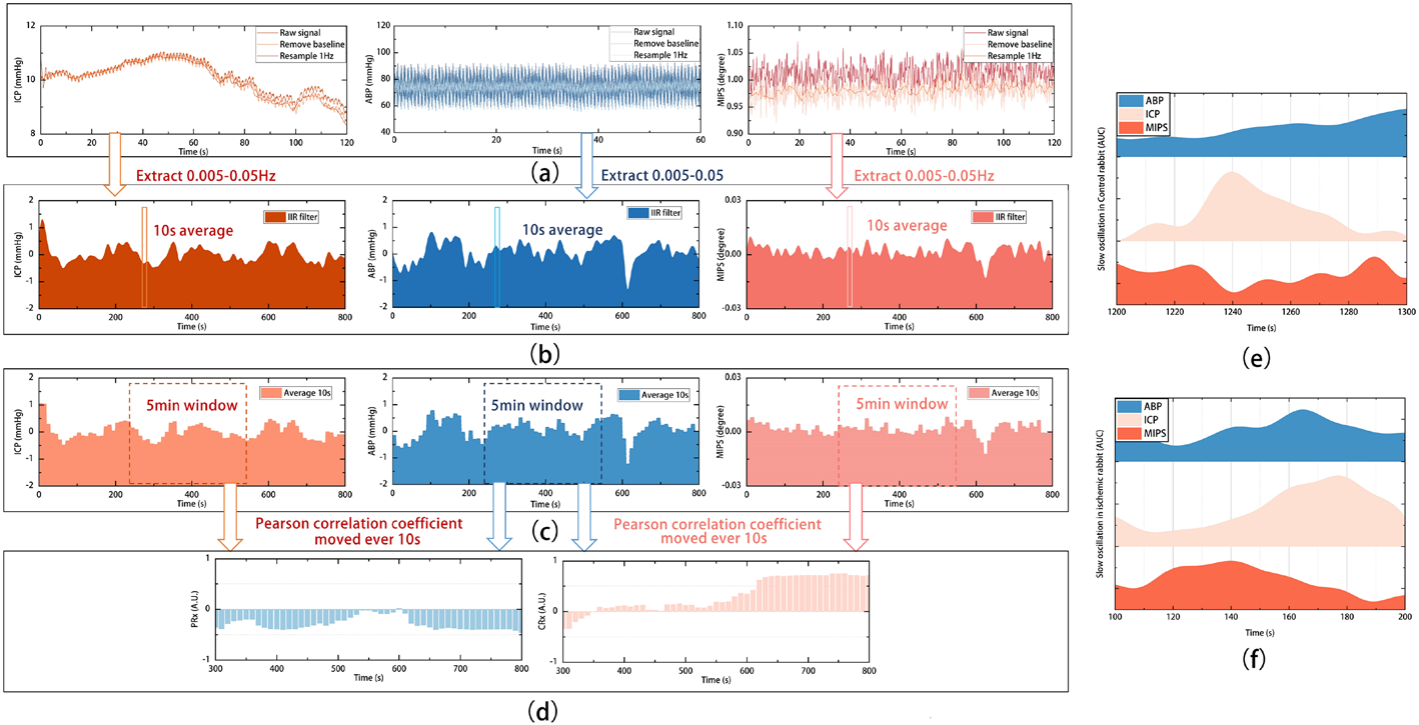
The CVAR index calculation process in the time domain (Control NO.8). **a** The preprocess signals. **b** Extracted slow frequency wave (0.005–0.05 Hz). **c** Average slow oscillations of ABP, ICP and MIPS every 10 s. **d** PRx and CRx calculated with moving Pearson’s correlation coefficient. Slow oscillations change in ABP, ICP and MIPS from **e** control rabbit (No. 8) and **f** ischemic rabbit (No. 4)

To improve the signal-to-noise ratio of the slow oscillations, we pre-processed the raw signals to remove high-frequency interferences such as respiration and heartbeat, extracted the slow oscillations components of ABP, ICP and MIPS, and calculated the PRx and CRx, as shown in Fig. 4. First, the original signals were down-sampled to 1 Hz after baseline drift was removed (Fig. 4a). Then, the low-frequency components below 0.05 Hz were extracted by a low-pass filter with a cut-off frequency of 0.05 Hz and a high-pass filter with a cut-off frequency of 0.005 Hz (Fig. 4b). After that, the extracted ABP, ICP and MIPS were averaged every 10 s (Fig. 4c). Finally, the Pearson correlation coefficients between mean values of ABP and ICP\MIPS were calculated using a 5-min time window repeated every 10 s to get the CVAR index (Fig. 4d).

The slow oscillation components were extracted from the ABP, ICP and MIPS signals collected simultaneously from the experimental rabbits, as shown in Fig. 4e, f. When cerebral ischemia is caused by reduced intracranial blood flow in rabbits, CVAR varies the slow oscillations of ICP and MIPS to maintain constant blood flow. In control rabbits, when ABP is increased, ICP does not passively increase in response to ABP in Fig. 4e; instead, when ischemia is present, ICP passively increases with arterial pressure, while MIPS inversely decreases in Fig. 4f. The phase difference between the slow oscillations in the ABP, ICP and MIPS signals is altered by cerebral ischemia. This phase difference is related to CVAR and can be evaluated by calculating the correlation coefficient between the excitation spontaneous slow oscillations in ABP and the response oscillations in ICP or MIPS.

Taking the control rabbits as a baseline, the percentage change of the mean value of slow oscillations components in ICP, ABP and MIPS values in the ischemic groups are shown in Fig. 3c. The mean value of ABP and ICP was increased and mean MIPS was negative increased after ischemia. The amplitude oscillation ($\left|\mathrm{Vmax}-\mathrm{Vmin}\right|$) of slow oscillations were expressed as dABP, dICP and dMIPS. Among these slow oscillation components, but the amplitude oscillation of MIPS decreased as the change of ICP.

### CRx between the cerebral ischemic group and control group

**Fig. 5:**
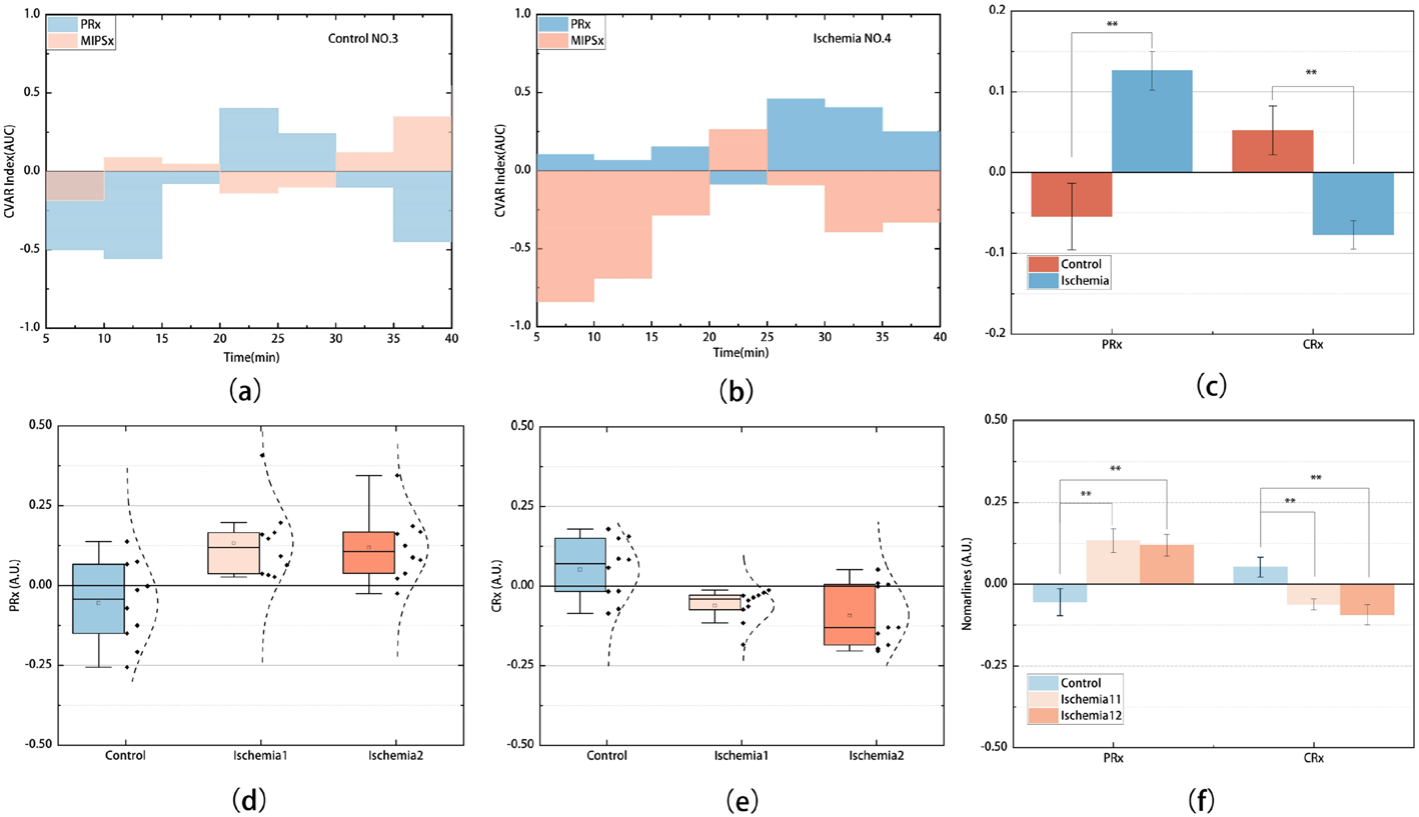
Comparison of PRx and CRx from the cerebral ischemia group and control group. PRx and CRx **a** of the control rabbit (No.3) and **b** of the ischemic rabbit (No.4). **c** The histogram of mean PRx and CRx comparison for the ischemia group (including ischemia1 and ischemia2) and control group. The box plot of **d** mean PRx and **e** mean CRx for the three groups. **f** The histogram of mean PRx and CRx for the three groups

The value of PRx and CRx (every 5 min) from the cerebral ischemia rabbit and control rabbit were compared in Fig. 5a, b. Generally, the CRx values oscillated inversely with the PRx values. In the control rabbit, the PRx oscillated mostly below zero and the corresponding CRx oscillated above zero, indicating a normal CVAR. In the ischemic rabbit, PRx oscillated above zero and CRx oscillated below zero most of the time, indicating impaired CVAR.

The CRx and PRx of each sample were averaged. 30 pairs of indexes were included in the statistics, 10 for the control group, 10 for ischemic group one (first 40-min period after ischemia), and 10 for ischemic group two (40 to 80-min after ischemia). The mean CRx and PRx of the three groups are shown in Fig. 5d, e. The mean PRx was -0.055 (SD = 0.130) in the control group, indicating that ICP was not directly driven by ABP and thus CVAR was intact, and the mean PRx was 0.133 (SD = 0.115) in the cerebral ischemia group 1, indicating impaired CVAR due to a passive relationship between ABP and ICP. On the other hand, the mean CRx changed from 0.052 (SD = 0.096) in the control group to -0.061 (SD = 0.017) in the ischemia group 1.

The independent sample t-test was used to analyze the difference of the mean PRx and CRx among groups. There were significant differences in CRx (*t* = 3.691, SE = 0.035, *p* = 0.002, 95% CI [0.054 to 0.204]) and PRx (*t* = − 3.800, SE = 0.048, *p* = 0.002, 95% CI [− 0.282 to − 0.080]) between the control group and the ischemic groups (Fig. 5c). There was no significant difference in CRx (*t* = 0.901, SE = 0.035, p = 0.379, 95% CI [− 0.042 to 0.106]) and PRx (*t* = 0.289, SE = 0.049, *p* = 0.776, 95% CI [− 0.089 to 0.118]) between the two ischemic groups (Fig. 5c, f). Although the statistics showed no statistical difference between the two ischemic groups, the mean PRx (Mean = 0.119, SD = 0.105) and CRx (Mean = − 0.093, SD = 0.098) were reduced in the ischemic group 2.

### The comparison of PRx and CRx for CVAR assessment

**Fig. 6:**
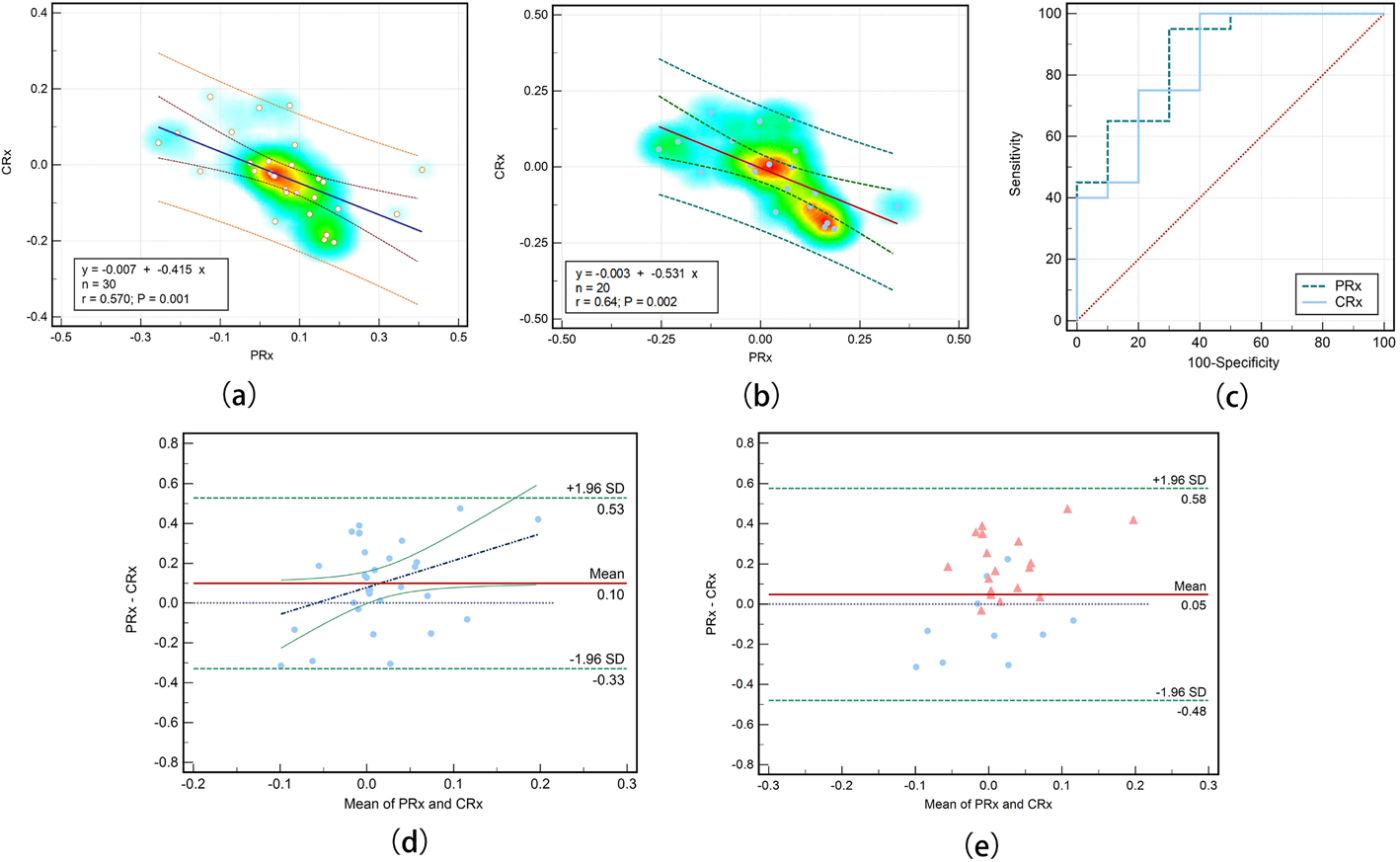
Relationship of PRx and CRx for CVAR assessment in early ischemia. Linear regression diagram of **a** 30 samples and **b** 20 samples. **c** ROC curve of CRx and PRx with control group vs. ischemia groups. **d** Bland–Altman plot and **e** Bland–Altman plot with multiple measurements per subject

Correlation analysis showed that CRx and PRx were significantly negatively correlated with a correlation coefficient r = − 0.570 (p = 0.001, 95% CI [− 0.772 to − 0.264]). The linear regression analysis with the mean CRx and PRx of all 30 samples is shown in Fig. 6a. The two indices were linearly correlated in the early phase of ischemia. The relationship between the two indices met the equation: $\mathrm{CRx}=-0.415\mathrm{PRx}-0.007({\mathrm{R}}^{2}=0.325)$. Linear regression analysis with the mean values of CRx and PRx in the 20 samples (control group and ischemia group 2) is shown in Fig. 6b. The fitting slope increased to -0.531 (*p* = 0.002) with a correlation coefficient r = − 0.64 (*p* = 0.002). Figure 6d, e shows the Bland–Altman plots for the two indices, showing the minimal difference and high consistency between the two indices for CVAR assessment in early ischemia. The mean difference between PRx and CRx was 0.100 (*p* = 0.018 < 0.05, 95% CI [0.018 to 0.182]) and the coefficient of repeatability of the two indices was 0.620 (Fig. 6d). Figure 6e shows that the arithmetic mean of the Bland–Altman plot with multiple measurements per subject is only 0.048.

Finally, we investigated the sensitivity and specificity of the two indices to discriminate cerebral ischemic rabbits from the control group. The area under the ROC curve of PRx is 0.865 (SE = 0.073). The sensitivity and specificity of PRx for identifying cerebral ischemia are 95% and 30%, respectively. The area under the ROC curve of CRx is 0.835 (SE = 0.084). The sensitivity and specificity for cerebral ischemia identification were 75% and 20% respectively for CRx, as shown in Fig. 6c.

## Discussion

In this study, based on the MIPS method, we investigated a novel conductivity reactivity index—CRx to monitor and assess CVAR at the early stage of cerebral ischemia in rabbits without head contact. The results showed that there was a significant difference in CRx between ischemic rabbits and control rabbits (*p* = 0.002), and the ischemic rabbits could be distinguished from the normal rabbits with an area under the ROC curve of 0.835, which preliminarily verified the feasibility of CRx in assessing CVAR injury in the early phase of ischemia. In the early ischemic period, the CRx and PRx were linearly related, with a minimal difference and high consistency for CVAR assessment. The study also showed that there was no significant difference in PRx and CRx between the two groups of cerebral ischemia, implying that the CVAR injury did not recover, whereas CRx decreased in the ischemic group 2, suggesting that there might be more CVAR impairment with time.

There is currently no gold standard indicator for CVAR assessment, but PRx has been widely used as a research standard. Here, we also chose PRx as a standard index and established relationships between the novel CRx and PRx. The results showed that the non-invasive CRx was significantly negatively correlated (*r* = − 0.570, *p* = 0.001) with the traditional invasive CVAR index-PRx. The negative correlation between the two indices was strengthened in ischemia group 2(*r* = − 0.642, *p* = 0.002). This may result from the fact that as the ischemic develops, some rabbits experience other lesions such as intracranial edema. With the result of CSF compensation, intracranial conductivity increases and MIPS decreases, so the negative correlation between the MIPS signal and the ICP signal increases. This may predict more severe injury. At the same time, the ROC curve also showed that the ability of CRx index to indicate injury increased slightly over time.

It is known that when the CVAR is intact, ABP slowly increases inducing vasoconstriction; then ICP decreases and the correlation coefficient (PRx) between the slow oscillations of ABP and ICP is negative. However, when CVAR is impaired, the increase in arterial pressure leads to passive vasodilation, which increases cerebral blood volume and ICP, yielding a positive correlation coefficient (PRx). In this study, the response slow oscillations in ICP do not increase constantly with increasing ABP excitation in the control group, which means that ICP is not directly driven by ABP and the CVAR is intact. As there is a poor correlation between the slow oscillations of ICP and ABP, the average correlation coefficient is negative (PRx = − 0.055 < 0). During cerebral ischemia, slow oscillations of ICP increase synchronously with ABP (Fig. 4f), and the correlation coefficient is positive (PRx = 0.133 > 0), indicating that CVAR was dysfunctional. These findings are consistent with previous research. On the other hand, the MIPS slow oscillations have a similar phase to the ABP slow oscillations in the control group and the decreased phase correlation with the ABP slow oscillations in the ischemic group. This opposite relationship of CRx to PRx (Fig. 5) is mainly due to the inverse phase variation of the MIPS signal compared to the ICP signal.

CRx was calculated as the correlation coefficient between the slow oscillations of ABP and MIPS (0.005–0.05 Hz). The MIPS slow oscillations associated with CVAR have a similar amplitude spectrum to ICP, but appear to be out of phase in early ischemia. MIPS is associated with cerebral blood volume and ICP [34, 37]. As the conductivity of Cerebrospinal Fluid (CSF) (2.002 S/m) is higher than that of blood (1.097 S/m), blood vessel wall (0.345 S/m), grey matter (0.292 S/m), white matter (0.158 S/m), dura (0.544 S/m) [42], MIPS is mainly influenced by CSF before the completion of CSF compensation in early ischemia. Physiologically, when intracranial pressure is increased, cerebrospinal fluid is pushed out of the cranial cavity, causing the global intracranial conductivity to decrease, resulting in an opposite decrease in MIPS, and when intracranial pressure is decreased, cerebrospinal fluid flows into the cranial cavity, causing the global intracranial conductivity to increase, resulting in an opposite increase in MIPS. These inverse slow oscillations of MIPS with ICP caused MIPS to track the slow oscillations of ABP in the control group (Fig. 4e) and to change inversely with ABP (Fig. 4f) in ischemic rabbits, which also caused the novel CRx showing the opposite variation to the invasive PRx (Fig. 5).

Here MIPS is considered to be a surrogate of CBV for CVAR monitoring. As the magnetic field of the MIPS method traverses the whole brain without the skull barrier, it can cover the small blood vessels which are the main contributors to CVAR and distributed throughout the brain [43]. The CBV changes in the small blood vessels in response to the slow oscillations of ABP will change the bulk conductivity of the brain. The electromagnetic parameter MIPS is not only affected by the intracranial local pressure but also affected by the proportion of intracranial tissue components, and the volume and location of diseased tissues. The CRx may therefore be a novel electromagnetic index to describe the properties of the CVAR. It has some differences from the mechanical parameter PRx but may provide more information on the brain using a multi-frequency or multi-coil magnetic induction system.

**Fig. 7:**
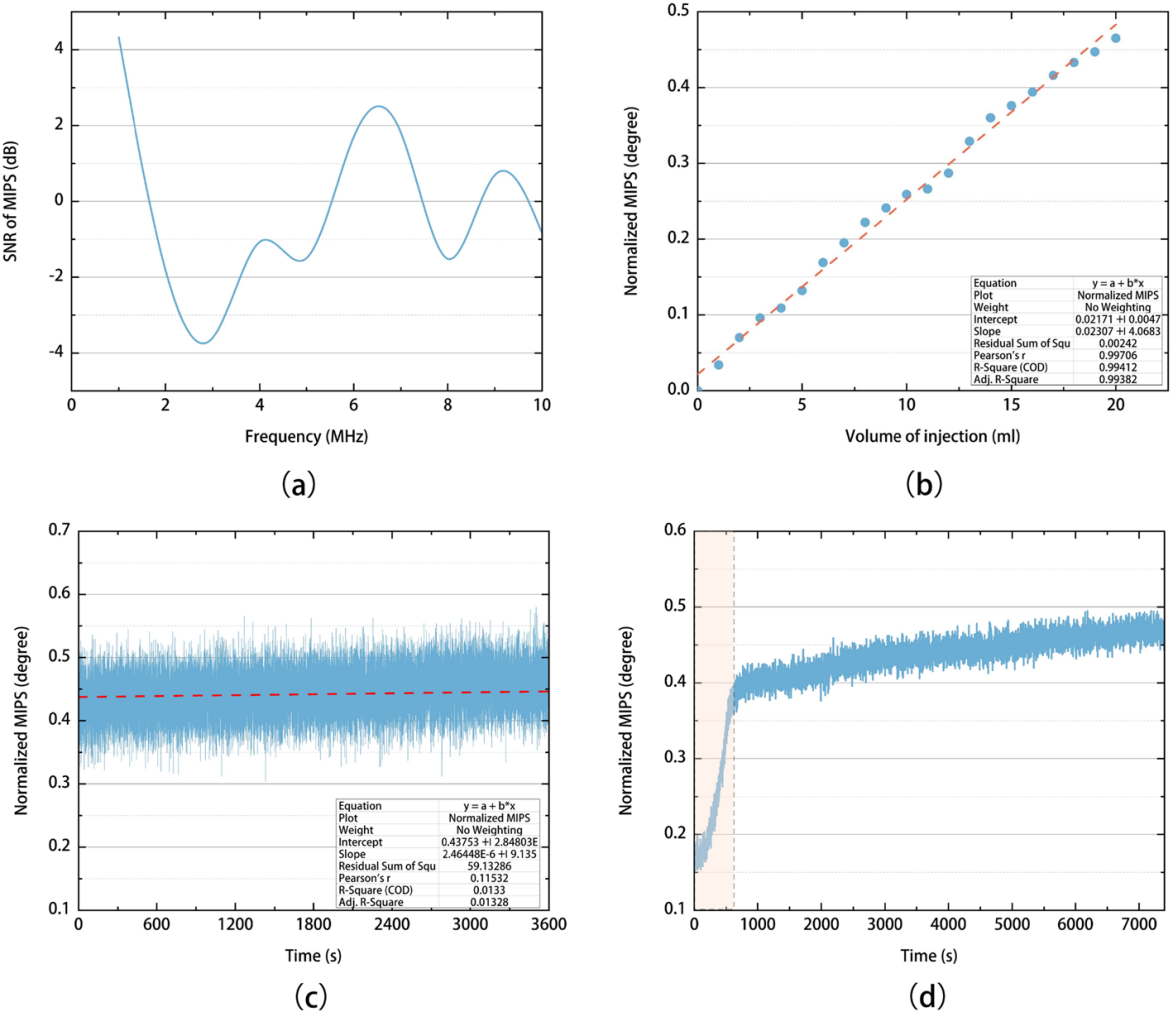
Performance of the experimental system. **a** Signal-to-noise ratio of MIPS with frequency. **b** The MIPS values with different injection volumes. **c** The system measurement across the coils in air as a function of time. **d** MIPS variation when injecting 10 ml of 9% saline with a syringe pump (the shaded interval is the injection process)

The CRx has been demonstrated to be able to monitor CVAR in the early phase of ischemia without contact to the head of the animal. The CRx index was positive in normal (CRx = 0.119 > 0) but negative in early ischemia (CRx = − 0.093 ≤ 0). This implies that the negative CRx values may indicate disturbed CVAR function. According to the linear regression between mean CRx and PRx shown in Fig. 7a, the threshold of CVAR injury indicated by the CRx index was -0.066, approximately zero. However, the accurate CRx threshold still needs to be determined experimentally.

Non-invasive technologies such as TCD and NIRS are also being investigated as alternatives to PRx [44]. TCD uses CBF velocity in the large vessels as a surrogate of CBF. There is a significant correlation between the PRx and the monitoring indices based on TCD (*r* = 0.6 to 0.78) [20, 22, 45, 46], but it is limited by operator experience, acoustic windows requirement, and probe displacement, which reduces the duration of continuous recording. Compared to TCD, CRx reflects the volume changes in the blood vessels of the whole brain and also the changes in cerebrospinal fluid affected by pressure, and CRx head sensors do not require touching the head and the magnetic field is not shielded by the skull. Diffuse correlation spectroscopy (DCS) is a promising optical technique for microcirculatory CBF monitoring in the cerebral cortex. There is a strong correlation between DCS and TCD-measured gain (*r* = 0.76) and phase (*r* = 0.65) in stroke patients [47]. NIRS detects local cerebral oxy/deoxy haemoglobin to get an oxygenation index to monitor CVAR. NIRS index has a correlation with the PRx (*r* = 0.4 to 0.6) [45, 48], but it is influenced by uncertain systemic factors. The novel CRx is significantly negatively correlated with PRx (*r* = − 0.640). Although currently also affected by artifacts, CRx is directly associated with intracranial volume and conductivity, reflecting the electrical properties of the whole brain, and is measured deeper than NIRS in a transcranial way. Impedance indices using trans-ocular brain impedance to assess CVAR. Brain impedance was recorded using electrocardiographic electrodes placed on the closed eyelids with an alternating current (0.4 mA) [30]. CRx measures deeper than trans-ocular brain impedance and without current coupling between the electrode and contacted skin. Compared to these non-invasive indices, the CRx is contactless on the head, global, depth and easy to operate. Our MIPS system had less phase deviation (± 0.033°) and higher resolution than the electromagnetic detection system used for CVAR (± 0.015°) in previous research [38]. However, spontaneous oscillatory BP stimulation has fewer variations and slightly larger standard deviations than other External stimulation strategies, which can be compensated by long-term monitoring. But it is worth mentioning that using spontaneous slow oscillations in ABP as the excitation, CRx does not cause discomfort and low risk to patients.

## Limitation

There are several limitations in this study. This study currently only used an animal model of diffuse cerebral ischemia to demonstrate the feasibility of the CRx to assess CVAR. It is unclear whether the relationship between CRx and PRx in other ischemic models can support the present finding. In the next stage, an animal model of local cerebral ischemic stroke will be established for CVAR assessment with CRx. In addition, studies have assessed the CVAR in the early ischemic phase, but when ischemia become more severe, how CRx change with different degrees of ischemic injury is unknown. As we know, the brain is a complex dielectric, changes in any tissue component can affect the conductivity of the whole brain, and the effect of complications such as cerebral hemorrhage and cerebral edema on CRx must be taken into account. The next step will investigate the relationship between CRx and the severity of the ischaemic injury by contrast with imaging experiments with a large sample size. Finally, this CRx measurement still uses invasive ABP monitoring. An alternative non-invasive measurement of arterial pressure using MIPS needs to be explored. The new generation CRx system will be established for non-invasive CVAR monitoring in humans.

## Conclusion

In this study, we explored the feasibility of a conductivity reactivity index-CRx to assess CVAR based on the noncontact MIPS method. The animal experimental results preliminarily demonstrated that the CRx can be used to monitor CVAR and identify CVAR injury in early ischemic conditions. In the future, the CRx has the potential to monitor and assess CVAR in a non-contact, bedside, global and natural way and be used for early alert of CVAR injury in ischemic stroke patients.

## Methods and materials

### Measurement system

The experimental system of this study consists of a MIPS detection system and a physiological signal recorder (Power Lab 8/35, AD Instruments Inc., Sydney, New South Wales, Australia) with two bridge amplifiers (FE221) and two physiological pressure transducers (SP844, MEMSCAP Inc., Research Triangle Park, Durham, USA) for ICP and ABP monitoring (Fig. 2).

The MIPS detection system consists of a MIPS sensor placed around the head, a signal generator (AFG3252 Tektronix Inc, Beaverton, Oregon, US) and an NI PXI acquisition control system embedded PXIe 5124 acquisition card and PXIe 8133 control card (National Instruments Inc, Austin, Texas, US). The MIPS sensor is constructed as coaxial parallel double coils [40, 49–51], comprising two electromagnetic coils (Fig. 2b). One coil serves as the emitter and the other as the receiver. Both coils were wound with the same number of turns (10 turns) and radius (5.2 cm) of 32 AWG copper wire (1 mm diameter) at both ends of a plexiglass tube, 10 cm apart. The emitter is connected to the signal generator and the receiver is connected to the signal acquisition card via the radio frequency connection cables. As the transcranial electromagnetic waves undergo a phase shift, the comparison of the voltages in the emitter and receiver could reflect the changes in the conductivity of the bulk brain tissue in response to the oscillations on the ABP.

An alternating current (5 Vpp, 6 MHz) from the signal generator is injected into the transmitter to generate an alternating primary magnetic field (B). When the primary magnetic field is disturbed by changes in volume or conductivity of the complex brain components, the magnetic field perturbation is measured by the phase shift of the voltage between the two coils, i.e., the MIPS. The acquisition card collects voltages in the two coils with 50 Ω input impedance and 100 MHz sampling frequency. Custom LabVIEW- based software is used to control synchronous acquisition and calculate phase shift between two acquisition voltages. The results of the MIPS system test are shown in Fig. 7. The MIPS data was obtained at 10 points per second, and synchronous ICP and ABP were monitored by the physiological signal recorder at 20 Hz sampling frequency.

The signal-to-noise ratio of the pulsation signal was measured at magnetic field excitation frequencies of 1 to 10 MHz (Fig. 7a), and finally the excitation frequency of 6 MHz was chosen for this study. the pulsation signal was simulated with a peristaltic pump (ZNB-XY1, CHINA) and one silicone tube through the sensor. The signal-to-noise ratio shown in Fig. 7a is only used to determine the excitation frequency of MIPS system. The signal-to-noise ratio between the slow waves and noise is about 20 dB in Fig. 3.

To test the MIPS monitoring system, the physical model of vascular pulsation was simulated and measured in an injection experiment using a syringe pump (LSP 01-1A, Baoding Ditron Electronic Technology Co., Ltd, Baoding, Hebei, China). One-hour MIPS signal was recorded for the phase shift of the MISP system (Fig. 7c) without the subject. The phase drift is 0.009° and the recorded MIPS signal is 0.444 ± 0.033° (Mean ± SD). The linearity and sensitivity of the MIPS system were measured by saline injection experiments. A beaker was placed in the center of the magnetic induction coil and then 20 ml of 9% saline solution was injected into the beaker in 20 aliquots of 1 ml each. The mean MIPS over 5 min measured after each injection are plotted in Fig. 7b. The results show that the linear correlation coefficient and sensitivity of the system were 0.997 and 0.023°/ml respectively. This is the resolution capability of the MIPS system. Figure 7d shows the MIPS injecting 10 ml of 9% saline using the syringe pump and the subsequent MIPS values. The change in MIPS caused by the injection, marked by the shading, differs by an order of magnitude from the subsequent MIPS for a constant volume.

### Animal model and data collection

The animals involved in this study were approved by the Animal Experiment Ethics Committee of the Army Medical University. Protocol number of the approval: AMUWEC2019237. Registered 11th Match 2019. The animal experiments were conducted following the Declaration of Helsinki and the guidelines issued by the International Association for the study of Pain (IASP). Twenty rabbits (2.2 ± 0.3 kg) were obtained from the Animal Experimental Center of Army Medical University and randomly divided into a cerebral ischemia group (n = 10) and a control group (n = 10).

This study used bilateral common carotid artery ligation to establish a rabbit model of cerebral ischemia [52]. All the rabbits were anesthetized by intravenous injection of uratan solution (25%, 5 mL/kg) and intramuscular injection of atropine sulfate (0.5 mg/mL, 0.2 mL/kg). Each rabbit was treated with tracheostomy and intubation to ensure unobstructed breathing. Based on the Sawyer rabbit brain stereotactic atlas [53], a 1.6 mm flat-tip needle connected to the physiological pressure transducer (SP844) via an infusion extension tube was inserted into the left lateral ventricle (AP = 0, L = 4 mm, H = 5 mm) and sealed with dental cement. In the cerebral ischemia group, the rabbit's bilateral common carotid arteries were separated and then ligated at the start of monitoring. An arterial cannula filled with sodium heparin solution was intubated into the femoral artery of the rabbit's right hind leg, the other end of which was connected to another physiological pressure transducer (SP844). The control group operated in the same way except for the ligation.

The rabbit's head was placed in the center of the MIPS sensor (Fig. 2(b)). After common carotid artery ligation in ischemic rabbits, MIPS, ABP and ICP data were acquired simultaneously. The monitoring period of the experiment lasted 80 min. The acquired data were stored after manually removing artifacts and analyzed offline using MATLAB software (MathWorks, Inc., Natick, Massachusetts, US).

Subsequently, after removing the baseline drift, the ABP, ICP and MIPS signals were down-sampled to 1 Hz. The slow oscillation components in ABP, ICP and MIPS were extracted by a 3rd-order Butterworth low-pass filter with a cut-off frequency at 0.05 Hz and a 3rd-order Butterworth high-pass filter with a cut-off frequency at 0.005 Hz.

After preprocessing, the cerebral autoregulation index-PRx was calculated [54]. ABP and ICP were averaged every 10 s to obtain mean ABP (MAP) and mean ICP (MICP) to reduce the influence of heartbeat and respiration. Finally, the Pearson correlation coefficient between the mean ABP and MICP is calculated using a moving window of 5 min. This calculation process is repeated every 10 s with the same moving window. The conductivity reactivity index-CRx is calculated between mean ABP and mean MIPS in the same way.

To assess CVAR at different stages of early ischemia, data from each rabbit (80 min) were divided into 2 segments (40 min each). The analysis data from the control rabbits were limited to the first 40 min to match the ischemia group. Finally, 30 segments of data (40 min/segment) were included in the statistics, 10 for the control group, 10 for ischemic group one (first 40 min), and 10 for ischemic group two (second 40 min).

The CRx and PRx of these segments in the three groups were averaged. The independent sample t-test was used to analyze the difference of two indices between the three groups. Linear regression and Bland–Altman analysis were performed to analyze the relationship between CRx and PRx. The receiver operating characteristic (ROC) curves of CRx and PRx were plotted to evaluate the sensitivity and specificity for identifying ischemia CVAR in the early phase. SPSS 25 software (IBM Corp., Armonk, New York, USA) and MedCalc^®^ Statistical Software version 19.7.2 (MedCalc Software Ltd, Ostend, Belgium; https://www.medcalc.org; 2021) were applied for statistical analyses.

## Data Availability

All data generated or analyzed during this study are included in this published article.

## Abbreviations

CVAR: Cerebrovascular autoregulation
CRx: Conductivity reactivity index
PRx: Intracranial pressure reactivity index
CBF: Cerebral blood flow
CBV: Cerebral blood volume
ABP: Arterial blood pressure
CPP: Cerebral perfusion pressure
ICP: Intracranial pressure
TCD: Transcranial doppler sonography
NIRS: Near-infrared spectroscopy
MIPS: Magnetic induction phase shift
NICUs: Neurological intensive care unit
CSF: Cerebrospinal Fluid
ROC: Receiver operating characteristic
AUC: Area under curve
